# Cross-habitat utilization of fish in a tropical deltaic system as a function of climate variability and body size: Are mangroves fish nurseries in a tropical delta?

**DOI:** 10.1371/journal.pone.0308313

**Published:** 2024-08-16

**Authors:** David Alejandro Sánchez-Núñez, Efraín Viloria Maestre, Mario Rueda

**Affiliations:** 1 Dirección Académica, Universidad Nacional de Colombia Sede de La Paz, La Paz, Cesar, Colombia; 2 Instituto de Investigaciones Marinas y Costeras de Colombia “José Benito Vives de Andréis” (INVEMAR), Santa Marta, Colombia; National Cheng Kung University, TAIWAN

## Abstract

The temporal variability of fish habitat utilization is poorly understood in tropical deltaic systems due to high water turbidity, which limits visual censuses, and to the lack of long-term data incorporating climate variability events. We aimed to assess the influence of body size and El Niño Southern Oscillation (ENSO) variability on the cross-habitat utilization rate of 14 fish species of commercial relevance in the Ciénaga Grande de Santa Marta (CGSM). We estimated the utilization of mangroves and coastal lagoons based on relative catch frequencies from encircling gillnets used within a long-term catch monitoring program, and then tested for significant changes in each species’ habitat utilization as a function of body size and climate variability. Six species showed a high dependence on mangroves and four on coastal lagoons for most body size classes (including juveniles) and ENSO conditions. One species (*Elops smithi*) showed a high utilization of mangroves in some ENSO phases and body size classes, while three species showed a high utilization of both mangroves and coastal lagoons. Mangrove utilization by six species (*Megalops atlanticus*, *E*. *smithi*, *Centropomus undecimalis*, *Mugil incilis*, *Mugil liza*, and *Ariopsis canteri*) increased in larger body sizes at low depths, which usually occurs under dry ENSO conditions, when predatory risk is higher in coastal lagoons. Another species (*Caquetaia kraussi*) increased its mangrove utilization from the body size at which its feeding habits change. Mangroves and coastal lagoons are important nurseries and habitats for adults of the main commercial fish species in the CGSM. Seascape habitats and fringe/riverine mangroves must be conserved in tropical deltas to promote not only nurseries but also fish lifecycles.

## Introduction

The presence of mangroves is often linked to higher fish densities in different geographical locations and environmental settings [1–4]. Although the nursery function has been predominantly highlighted in most mangrove fish and fishery studies, the high utilization of sub-adults and adults has also been described in several fish species and mangrove settings [5, 6]. The nursery function of mangroves has been associated with the supply of shelter from predators, greater food availability, and the favorable microclimatic and hydrodynamic conditions for juveniles within mangrove roots [7, 8]. On the other hand, the increased utilization of mangroves and macrophyte vegetation by some species of larger fish has been linked to ontogenetic dietary shifts, higher mobility, and the supply of adequate shelter during daytime [6, 9–11].

Mangrove-fish relationships have been broadly studied in oligotrophic clear waters (*i*.*e*., Caribbean karstic areas), while fewer studies have been carried out in deltaic systems, which are usually subject to high nutrient loads and turbidity levels, and where seagrasses and coral reefs are limited or absent [3, 11–14]. The underrepresentation of deltaic mangrove settings in the literature linking mangroves and fishery is probably due to low visibility in these turbid waters, which hinder visual censuses, a cost-efficient method broadly used to analyze the habitat dependency and shifts of fish in mangrove environments with clearer waters [2, 15]. Since 40% of the world’s mangroves are deltaic [16], there is a clear knowledge gap in understanding mangrove-fishery relations and the flexibility of habitat utilization by fish in this dominant mangrove type, such as cross-habitat movements and utilization over entire lifecycles [5, 9, 17].

Assessing habitat utilization changes in estuarine systems as function of climate variability is a research priority for a better understanding of cross-habitat connectivity [5, 18, 19]. Studies relating climate variability and fish populations in tropical deltaic systems have focused less on habitat utilization and more on the effects of fish abundance [20–23]. In deltaic [24] and coastal settings [25, 26], species-specific ontogenetic habitat use patterns have been found within multi-habitat seascapes. Additionally, in non-estuarine mangrove systems, fish species have shown different degrees of habitat use flexibility as a response to spatial, seasonal, and interannual variations [27].

The ecoregion of the Ciénaga Grande de Santa Marta (CGSM) is one of the largest deltaic systems in the Greater Caribbean [28]. The CGSM is highly influenced by climate variability from the El Niño southern oscillation (ENSO), which drives changes in water levels, salinity, mangrove cover, and fish species composition and abundance [29–31]. On the other hand, the CGSM is a relatively well-studied system, where long-term fishery monitoring regarding catch and body size composition is carried out. In the CGSM, the traditional ecological knowledge clearly recognizes the value of mangroves for fisheries [24, 32–34]. Long fisheries datasets offer an opportunity to study the cross-habitat utilization of fish resources in this major deltaic system of the Caribbean.

This study aimed to assess whether the main estuarine and freshwater fish species captured by small-scale fisheries in the CGSM change their relative habitat utilization according to their body size and in response to climate variability. We anticipated finding species-specific habitat preferences for different body sizes, as observed in other seascape environments [24, 26]. Based on the well-known nursery function of mangroves, we hypothesized that individuals in juvenile life stages would dominate fishery catches inside the mangroves, with larger size classes found in coastal lagoons. In addition, we anticipated that habitat utilization would be affected by climate variability, as has been identified in other mangrove settings [27].

## Materials and methods

### Study area

**Fig 1:**
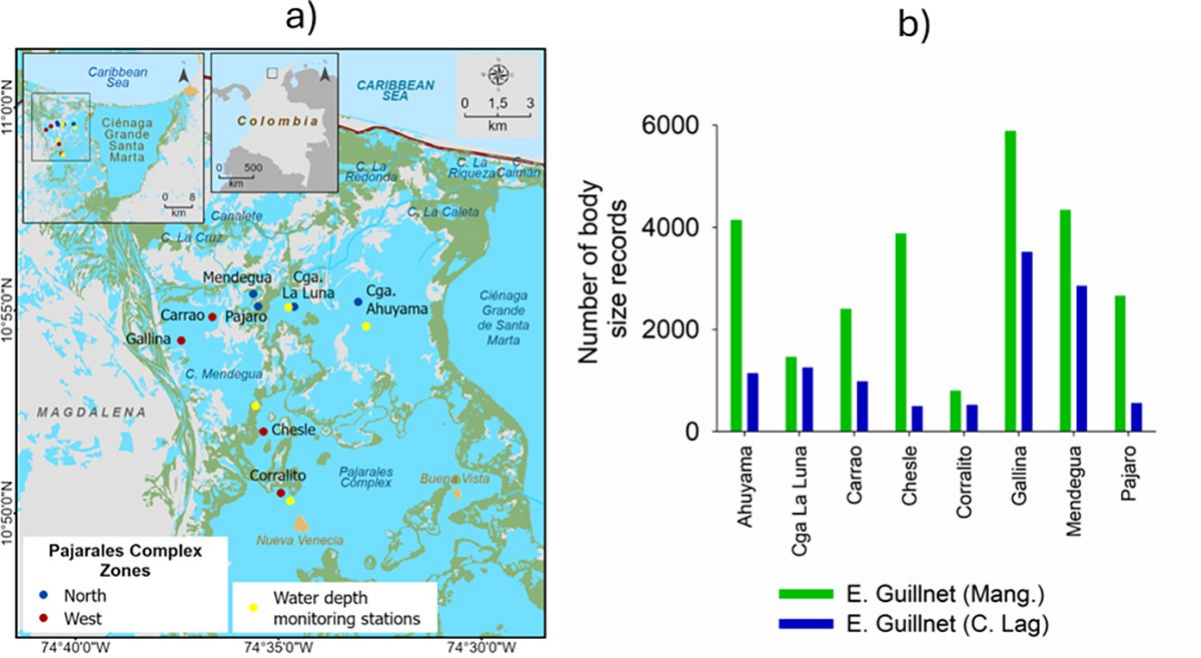
Study area and fishing records per habitat. (a) Location of fishing grounds and water depth monitoring stations; (b) body size records by fishing ground. Panel (a) was reprinted from INVEMAR under a CC BY license, with permission from original copyright, 2024.

The CGSM is located between the coordinates 10°40’ and 10°59’N and 74°15’and 74°38’W [35], as shown in Fig 1A. Its climate is tropical semiarid, with an average temperature of 27.5°C and average precipitation and evapotranspiration values of 400 and 1431 mm year^-1^, respectively [36]. The Fundación, Aracataca, and Sevilla Rivers drain the Sierra Nevada de Santa Marta and are directly connected to the CGSM’s main coastal lagoon, while the Magdalena River is connected to the system through the Clarín, Aguas Negras, and Renegado Channels [28, 37].

The CGSM has the largest mangrove extension (35,379 ha in 2019) and the largest coastal lagoon in the Colombian Caribbean (44,010 ha in 2018) [38, 39]. Its fishery is one of the most important in Colombia, supporting approximately 4000 fishermen, who catch more than 50 species of fish, crustaceans, and mollusks using several fishing gears and methods. Between 1994 and 2020, the CGSM fisheries monitoring program estimated annual catches of 4178–9269 t and an average fisher monthly income of 821–144 million COP. On the other hand, the climate variability caused by the ENSO has been identified as the main driver of changes in salinity and in the abundance and composition of species targeted for fishing [29].

On an interannual basis, CGSM fishery exhibits a highly variable natural productivity, and, despite its ecological, economic, and social importance, it shows signs of overfishing. Apart from fishermen, the main fish predators in this region are piscivorous birds. This trophic guild is the most predominant, with 23 species and 47% of bird detections, including herons, egrets, pelicans, cormorants, gulls, terns, storks, kingfishers, ducks, and ospreys [40].

The CGSM experienced a strong environmental degradation between 1956 and 1995, losing 56% of its original mangrove coverage (51,150 ha), due to the construction of a road parallel to the coastline, in addition to a road and dikes in the Magdalena River floodplain, which restricted tidal and freshwater connectivity [41]. A rehabilitation project called *PROCIENAGA* reconnected the CGSM to the Magdalena River delta. This was done by constructing three river diversion channels between 1996 and 1998 in sites that previously connected the river with coastal lagoons during floods [42]. This hydrological rehabilitation project along with continuous channel maintenance, have recovered an area equivalent to 33% of the original mangrove coverage [39]. Mangrove coverage fluctuations have also occurred as a consequence of ENSO climate variability, with dry periods (El Niño phases) driving coverage losses and wet periods (La Niña phases) driving coverage gains [31]. The CGSM’s area of fringe/riverine mangroves, subject to tidal and fluvial inundation, is approximately 941 ha, compared to the 77,461 ha occupied by coastal lagoons [43].

The Pajarales Complex, a system of coastal lagoons surrounded by mangroves, is located in the northwest of the CGSM ecoregion. Within this complex, there are two small stilt villages of fishermen, with a combined population of 2452 inhabitants [44]. Here, the salinity and dissolved oxygen concentration decline during the La Niña phase, in comparison with neutral ENSO conditions. These parameters increase during El Niño, as ENSO climate variability influences the freshwater flows from the rivers and channel diversions that feed the system [28, 45]. In the northern region of the Pajarales Complex, the average salinity (±SD) values are 8.0±1.2, 19.0±1.1, and 31.2±1.9 g L^-1^ during La Niña, neutral, and El Niño phases, respectively. Meanwhile, the western part reports 6.2±1.2, 13.3±1.1, and 26.1±1.7 g L^-1^ for the same phases. The oxygen concentrations reported by northern Pajarales are 7.6±0.2, 8.2±0.2, and 8.2±0.3 mg L^-1^, and those of the western region are 8.4±0.3, 8.9±0.2, and 9.0±0.3 mg L^-1^ (S1 Fig). In this region, strong salinity changes favor estuarine species fish during neutral and El Niño phases, as well as freshwater ones under La Niña conditions.

During the wet season, fringe and riverine mangroves, where encircling gillnets are used for fishing, experience daily tidal inundation and high water levels due to increased freshwater delivery from the Aguas Negras Channel into coastal lagoons. Fringe and riverine mangroves and their adjacent coastal lagoon share similar salinity levels. These ecosystems measure around 10 m in width and feature *Rhizophora mangle* as the dominant species.

### Fish species studied

The 14 most abundant commercial species in the CGSM were studied. Of these, eight are estuarine and six are freshwater species. All of them are native, except for the Nile tilapia, which was introduced in Colombia between 1970 and 1975 and invaded the Pajarales Complex in 1995 [23]. It was assumed that this species had already adapted to the complex by 2001 (*i*.*e*., the starting year of our dataset), given its high adaptability, reproductive success [46], and the time it had to adapt since its introduction in the Colombian Caribbean. Currently, the Nile tilapia is one of the species with the highest commercial relevance for artisanal fisheries. Between 2001 and 2019, it represented 15% of the catches in the Pajarales Complex. The feeding habits of the studied CGSM species are summarized in Table 1.

**Table 1:** Feeding habits of estuarine and freshwater species in the Ciénaga Grande de Santa Marta.

Species	Feeding habits	Dietary components	Sources
Estuarine			
*Megalops atlanticus*	Carnivorous	Fish and crustaceans	[47]
*Elops smithi*	Carnivorous	Fish, crustaceans, insects	[48]
*Eugerres plumieri*	Omnivorous	Mollusks, annelids, crustaceans, insects, plant material	[49]
*Ariopsis canteri*	Mainly Carnivorous	Fish and crustaceans (mainly), plant material, insects	[50]
*Centropomus undecimalis*	Carnivorous	Fish and crustaceans	[51]
*Mugil incilis*	Herbivorous, detritivorous	Phytoplankton, detritus, zooplankton	[50, 52]
*Mugil liza*	Herbivorous, detritivorous	Detritus, phytoplankton, zooplankton	[50, 52]
*Cathorops mapale*	Mainly Carnivorous	Fish (mainly), insects, crustaceans, plant material, mollusks, zooplankton	[50]
Freshwater			
*Oreochromis niloticus*	Mainly herbivorous	Phytoplankton (mainly), zooplankton, plant material, detritus	[50]
*Caquetaia kraussi*	Mainly Carnivorous	Fish (mainly), insects, vegetal materials, crustaceans, zooplankton	[50]
*Megaleporinus muyscorum*	Mainly herbivorous	Plant material (mainly), insects	[53]
*Prochilodus magdalenae*	Detritivorous	Detritus (mainly), phytoplankton	[54]
*Hoplias malabaricus*	Carnivorous	Fish (mainly), insects	[55]
*Triportheus magdalenae*	Carnivorous	Zooplankton (mainly), insects	[56]

### Catch data by habitat

The catch data used in this study originate from the fishing monitoring system (SIPEIN) deployed by the Colombian Institute for Marine and Coastal Research (INVEMAR) in the artisanal fisheries of the CGSM and were collected between 1994 and 2020 [57, 58]. The SIPEIN was developed to monitor fishing resources in the CGSM and records daily fishing catches three days a week [59]. In the Pajarales Complex of the CGSM, encircling gillnets with semicircles are independently used for fishing in two habitats: coastal lagoons and mangroves. While using this fishing gear, shallow waters are pounded to promote sediment suspension, thereby asphyxiating the fish [60, 61]. In coastal lagoons, encircling gillnets are deployed beyond 20 m (even hundreds of meters) from the mangrove edges [32]. 5–10 cm mesh is used in both mangroves and coastal lagoons, with mean sizes of 6.6 and 5.1 cm, respectively.

We used SIPEIN data on eight fishing grounds located in the north and the west of the Pajarales Complex from 2001 to 2019. In these areas, the use of encircling gillnets is commonplace (Fig 1B).

### Intensity of climate variability

The intensity of the cold and warm episodes of the ENSO was determined using the Oceanic El Niño Index (ONI) [62]. This index is estimated by the NOAA on a monthly basis, and values have ranged from -1.8 to -1.6 during strong La Niña episodes and up to 2.6 during the very strong El Niño of 2015. The fishing records were categorized based on climate variability conditions (El Niño, La Niña, and neutral) and the monthly ONI values [62]. The ENSO affects the regional and local rivers feeding the system in a similar way, with precipitation and water flows increasing during El Niño and decreasing during La Niña [28, 45]. Therefore, it was not necessary to discriminate between local and regional influences with regard to climate variability in the CGSM system.

### Water depth

The water depth in the Pajarales Complex during fish catching periods was determined by averaging the depths of four monitoring stations in the area (Fig 1A). On average, INVEMAR monitors each station for eight months per year [63], measuring the water depth *in situ* from a boat with a Secchi disk.

### Data analysis

#### Relative habitat utilization

The relative habitat utilization of fish species (***i***) in each of the two Pajarales Complex zones (***Z***) and each of the two studied habitats (***h***) was estimated for different body sizes (***bs***) and ENSO phases (***E***), by means of the relative catch frequencies (***Fr***). The catch frequency of a species in a habitat (***Fr***_***bs*,*i*,*h*,*E*,*Z***_), divided by the total catch frequency across both habitats (***FrT***_***bs*,*i*,*E*,*Z***_), indicates the probability of occurrence and, therefore, the relative habitat utilization (***Frh***_***bs*,*i*,*E*,*Z***_) (Eq 1). ***Fr***_***bs*,*i*,*h*,*E*,*Z***_ is relative to the total number of individuals of all species captured within each habitat, and ***Fr***_***bs*,*i*,*E*,*Z***_ is relative to the total number of individuals of all species captured in the two habitats. Based on the maturity size of each species in the CGSM [59] and the observed changes in catch frequency with body size between habitats, two to four main body size classes were identified for each species. The fishing records from the four fishing grounds in each zone of the Pajarales Complex were pooled together to carry out the aforementioned estimations (Fig 1B).

$${Frh}_{bs,i,E,Z}=\frac{{Fr}_{bs,i,h,E,Z}}{{FrT}_{bs,i,E,Z}}$$ (1)

In addition to estimating the relative habitat utilization for each ENSO phase, we also calculated this variable for each fish species (***i***) in the Pajarales Complex, according to the intensity of cold and warm ENSO episodes (*EI*) (Eq 2).

$${Frh}_{i,EI}=\frac{{Fr}_{i,h,EI}}{{FrT}_{i,EI}}$$ (2)

Although the fish stocks for each ENSO condition and year were unknown, it was assumed that the relative frequencies resembled the occurrence probabilities, thereby indicating the habitat utilization, given the temporal continuity of SIPEIN monitoring and the large dataset considered (36,817 body size records in the Pajarales Complex). In addition, the fishing power of the encircling gillnets was considered for all the species in the water column, owing to the shallow depth of the studied region (0.2–1.7 m), which allows deploying this fishing gear from the sediment bed of the water column [64].

#### Differences in habitat utilization

A Mann-Whitney U test was conducted with the purpose of identifying significant differences in the relative habitat utilization of fish species in mangroves and coastal lagoons, grouping all sizes and ENSO conditions.

#### Habitat utilization in response to size and climate variability phases

Two-way Scheirer-Ray-Hare (SRH) tests [65, 66] were employed to assess the influence of size and climate variability (factors) on habitat utilization. This analysis was carried out for each species and habitat. The relative habitat utilization values of each of the two studied Pajarales regions were used as replicates. Three species were analyzed separately: *C*. *kraussi*, *H*. *malabaricus*, and *P*. *magdalenae*. The largest body size class of *C*. *Kraussi* was absent in coastal lagoons during all ENSO phases and in all Pajarales zones, hindering the use of two-factor SRH tests with 3*3 factor levels. Instead, we conducted SRH tests with 2*3 factor levels for *C*. *Kraussi*. The other two species, *H*. *malabaricus* and *P*. *magdalenae*, were absent in both habitats during El Niño and La Niña in one of the Pajarales zones and in almost all size classes, also hindering the SRH tests. For these two species, we performed a Kruskal-Wallis test to independently assess the influence of size and climate variability on habitat utilization. The low number of replicates impeded *post hoc* comparisons of SRH treatments, so we conducted paired Mann-Whitney U test comparisons for each estuarine or freshwater species, in order to identify which habitat had a significantly higher utilization across different ENSO conditions and body size classes.

#### Habitat utilization in response to the intensity of climate variability and water depth

First, a Spearman correlation analysis was conducted to assess the relationship between each species’ average catch body size and relative habitat utilization (***Frh***_***i*,*EI***_) in response to the monthly ONI records [62]. For the species with significant correlations or whose habitat utilization significantly changed with size (see the SRH test described in the previous section), we performed a regression analysis of the water depth *vs*. the average catch body size per habitat. The water depth data were organized in intervals of 0.03 cm, and the body sizes present in each interval were averaged.

ENSO climate variability generates changes in the CGSM’s water depth [67]. To confirm the existence of a relationship between this variability and the water depth of the Pajarales Complex, we performed simple and multiple regression analyses of water depth as function of the ONI and the intra-annual seasonality, evaluated as the average time of the year in months. The Aguas Negras Channel has a marked intra-annual seasonality: its freshwater flows are 17–32 m^3^ s-^1^ between January and April, 39–58 m^3^ s-^1^ between May and August, and 50–80 m^3^ s-^1^ between September and December [68]. It is important to add that all statistical analyses were carried out using STATISTICA 10.0 and R 4.4.0.

### Ethics statement

INVEMAR is the scientific branch of the Colombian Ministry of Environment and Sustainable Development (MinAmbiente) entrusted with research and monitoring in coastal and marine ecosystems. CORPAMAG, the regional environmental authority of the study site, and MinAmbiente designated INVEMAR to monitor fish catches in the CGSM. In this vein, INVEMAR does not require a permit to access or conduct research in the study site. The SIPEIN records fish catches at the harbor to monitor fishing resources. Fish die mainly by asphyxiation due to the use of encircling gillnets. In this study, we were not involved in the sacrifice of fish. Research involving the monitoring of catches by fishermen for subsistence purposes does not require a permit from the research ethics committee of INVEMAR.

## Results

### Catch frequencies by habitat

**Fig 2:**
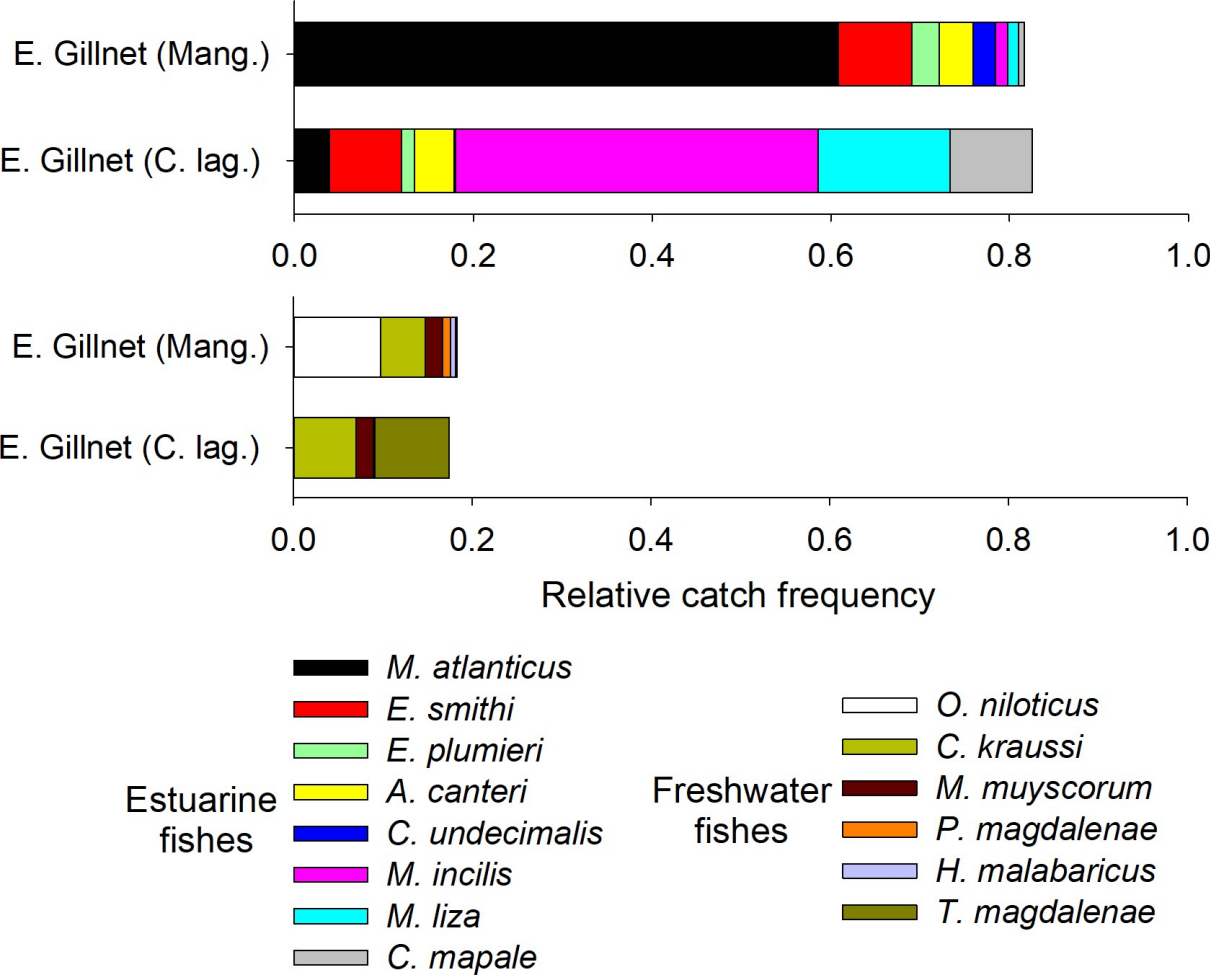
Relative catch frequency by habitat/zone for the main 14 estuarine and freshwater fish species in the Pajarales Complex of the CGSM.

The most frequent species in the mangroves were *M*. *atlanticus*, *O*. *niloticus*, and *E*. *smithi*, while, in the coastal lagoons, *M*. *incilis*, *M*. *liza*, and *C*. *mapale* exhibited high relative catch frequencies (Fig 2). Nine species had a significantly higher preference for mangroves, as well as four for coastal lagoons. One species did not show a significantly higher utilization of any habitat (Table 2).

**Table 2:** Mann-Whitney U test to assess the differences in the relative habitat utilization of the main commercial estuarine and freshwater fish species of the CGSM, including their body size ranges per habitat.

Species	Z-score	Body size range (cm)	
		Mangroves	Coastal lagoons
Estuarine			
*M*. *atlanticus*	4.1*[M]	14.0–80.5	14.0–56.0
*E*. *smithi*	2.6*(M)	15.0–56.5	18.5–53.5
*E*. *plumieri*	4.1*[M]	13.5–37.0	19.0–33.0
*A*. *canteri*	2.9*(M, CL)	17.5–49.5	18.5–42.5
*C*. *undecimalis*	5.1*[M]	15.5–65.0	31.5–49.5
*M*. *incilis*	-4.1*[CL]	14.5–40.5	15.0–39.5
*M*. *liza*	-5.1*[CL]	27.0–56.5	19.5–56.5
*C*. *mapale*	-4.1*[CL]	14.5–28.0	13.0–26.5
Freshwater			
*O*. *niloticus*	5.8*[M]	14.0–36.0	
*C*. *kraussi*	0.6^ns^(M, CL)	12.0–37.0	12.5–28.0
*M*. *muyscorum*	2.0*(M, CL)	18.5–42.5	19.5–39.5
*P*. *magdalenae*	4.5*[M]	20.5–45.0	24.5–30.5
*H*. *malabaricus*	4.1*[M]	19.5–40.5	37.5–41.5
*T*. *magdalenae*	-4.1*[CL]	14.5–23.5	13.0–26.0

*p < 0.05, ^ns^*p* > 0.05. Habitats with an average utilization greater than 35% are indicated in parentheses, and those with a value greater than 75% are indicated in square brackets. M: mangrove, CL: coastal lagoons.

### Water depth as function of climate variability

**Fig 3:**
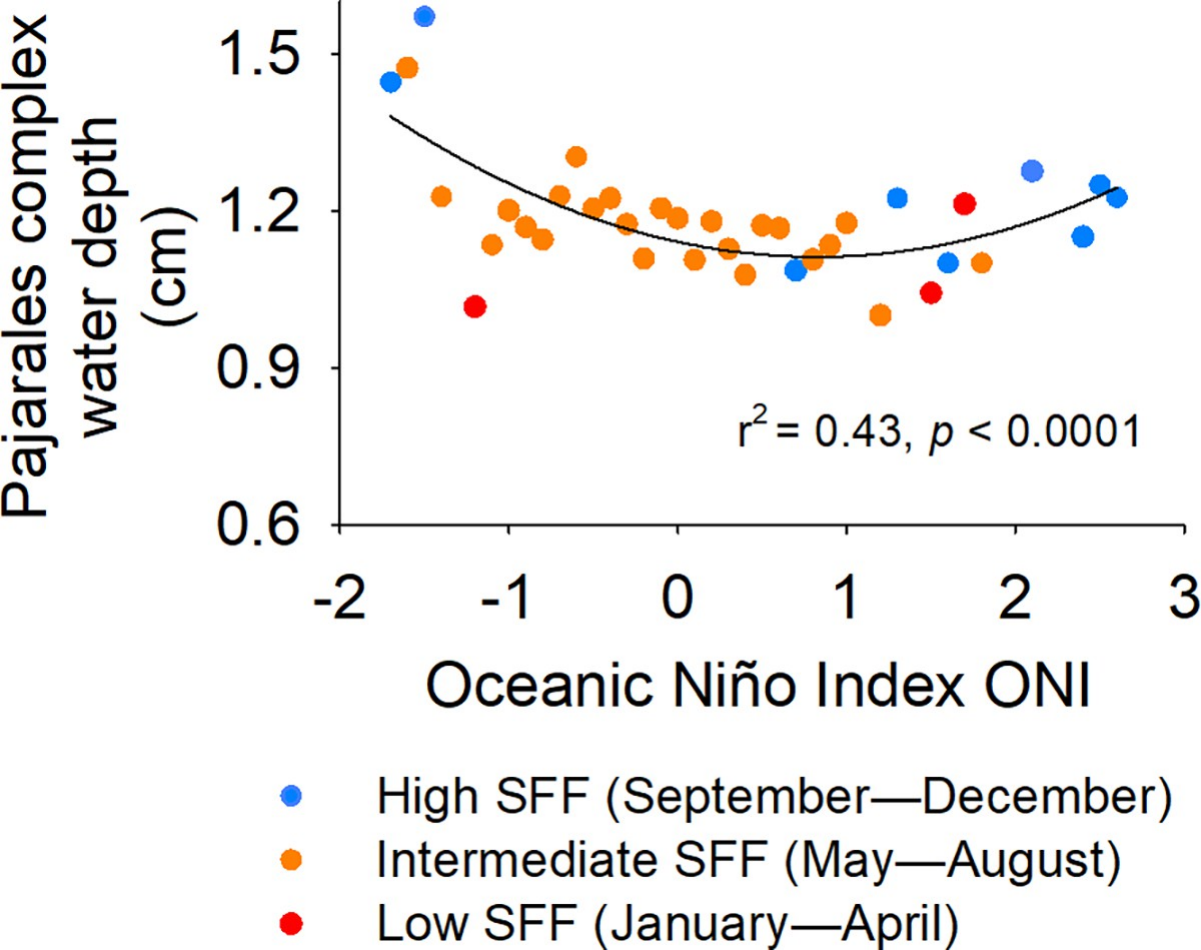
Water depth of the Pajarales Complex as a function of the ONI. SFF: seasonal freshwater flow.

The intensity of climate variability and the intra-annual seasonality collectively explained the water depth of the Pajarales Complex (r^2^ = 0.47, *p* < 0.01 for both independent variables), with climate variability explaining a relatively high degree of the water depth variability (Fig 3). Thus, stronger El Niño episodes and dry seasons generate lower water depths, while wet seasons and stronger La Niña phases entail higher depth values.

### Habitat utilization by estuarine fish species in response to size, climate variability intensity, and water depth

**Fig 4:**
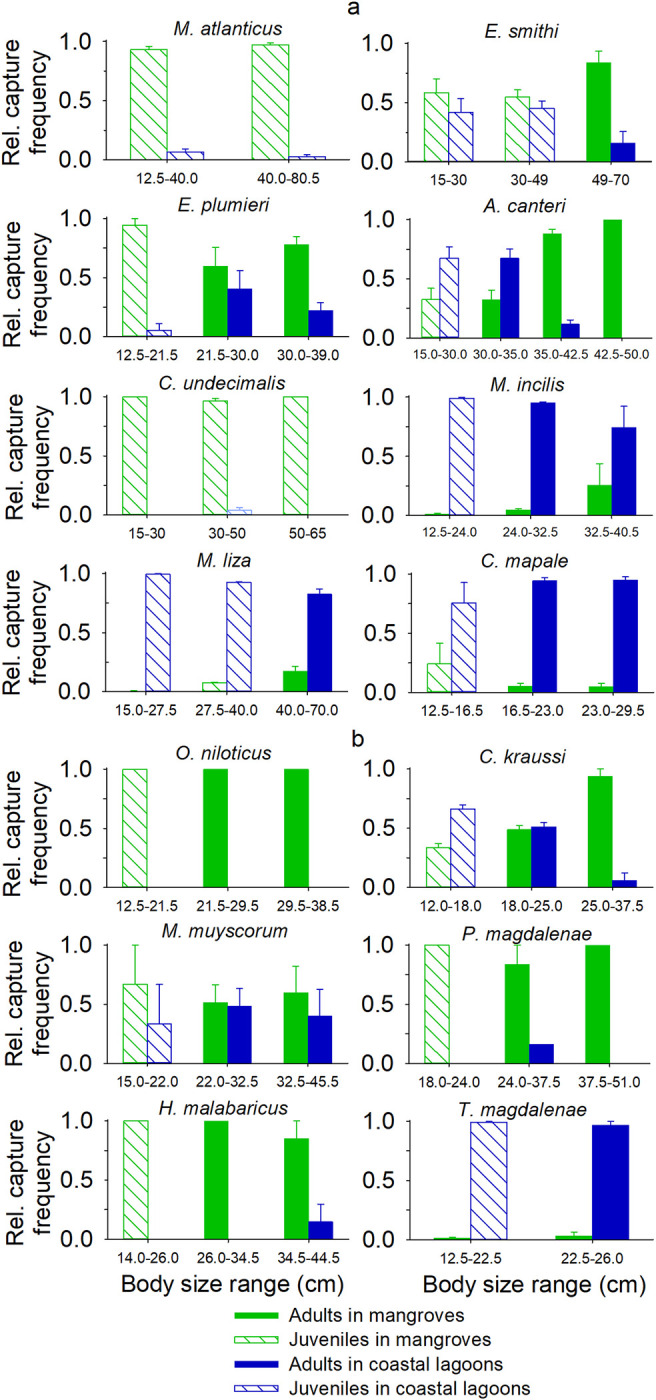
Relative habitat utilization for the body size classes of the fish species in the Pajarales Complex of the CGSM. The standard error in the relative catch frequency between ENSO phases is shown. (a) Estuarine and (b) freshwater fish species.

**Fig 5:**
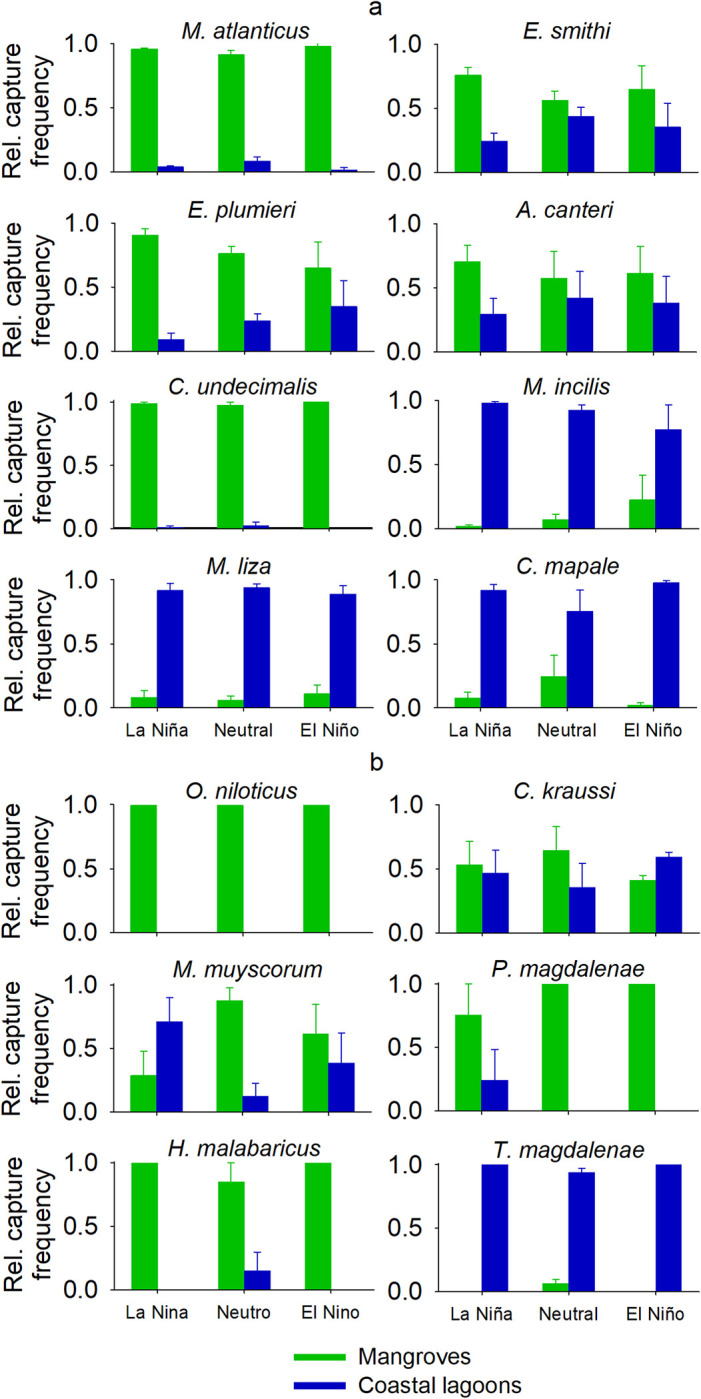
Relative habitat utilization across ENSO phases of the fish species of the Pajarales Complex in the CGSM. The standard error in the relative catch frequency between body size classes is shown. (a) Estuarine and (b) freshwater fish species.

The utilization of *M*. *atlanticus* and *C*. *undecimalis* in mangroves was significantly higher in comparison with that of coastal lagoons for all body size classes and ENSO conditions (Figs 4 and 5) (Mann-Whitney U, *p* < 0.05). The habitat utilization of both species significantly changed with size (Table 3).

**Table 3:** SRH test regarding the effects of ENSO phases (El Niño, neutral, La Niña) and body size classes on the habitat utilization of the main estuarine and freshwater commercial fish species of the CGSM.

Especies	Mangrove			Coastal lagoons		
	ENSO	Size	ENSOxSize	ENSO	Size	ENSOxSize
Estuarine						
*M*. *atlanticus*	4.6^ns^	6.7*	0.1^ns^	3.8^ns^	5.6^ns^	0.5^ns^
*E*. *smithi*	1.4^ns^	3.0^ns^	0.8^ns^	1.7^ns^	2.1^ns^	0.8^ns^
*E*. *plumieri*	1.7^ns^	3.0^ns^	0.5^ns^	3.0^ns^	3.6^ns^	0.9^ns^
*A*. *canteri*	0.4^ns^	44.7*	1.5^ns^	0.9^ns^	28.1*	1.2^ns^
*C*. *undecimalis*	1.8^ns^	6.2*	2.0^ns^	0.9^ns^	3.2 ^ns^	1.0^ns^
*M*. *incilis*	20.0*	36.2*	9.2*	13.2*	22.8*	6.2*
*M*. *liza*	0.2^ns^	9.1*	0.3^ns^	0.4^ns^	15.4*	0.8^ns^
*C*. *mapale*	3.0 ^ns^	0.8^ns^	1.8^ns^	3.6^ns^	0.1^ns^	1.5^ns^
Freshwater						
*M*. *muyscorum*	6.7*	1.2^ns^	3.2^ns^	10.3*	0.5^ns^	5.1*
*T*. *magdalenae*	39.1*	5.7^ns^	6.3*	6.4*	0.1^ns^	1.5^ns^
*C*. *kraussi 2E*, *3T*	4.5^ns^	32.4*	0.1^ns^	4.3^ns^	30.9*	0.6^ns^
*C*. *kraussi 3E*, *2T*	0.4^ns^	0.7^ns^	0.8^ns^	0.3^ns^	1.7^ns^	0.3^ns^
*H*. *malabaricus 3E*, *2T*	0.3^ns^	0.2^ns^	0.3^ns^	0.3^ns^	0.2^ns^	0.3^ns^

*p < 0.05, ^ns^*p* > 0.05. 3E, 2T: three ENSO phases and two body sizes compared. 2E, 3T: two ENSO phases and three body sizes compared.

**Fig 6:**
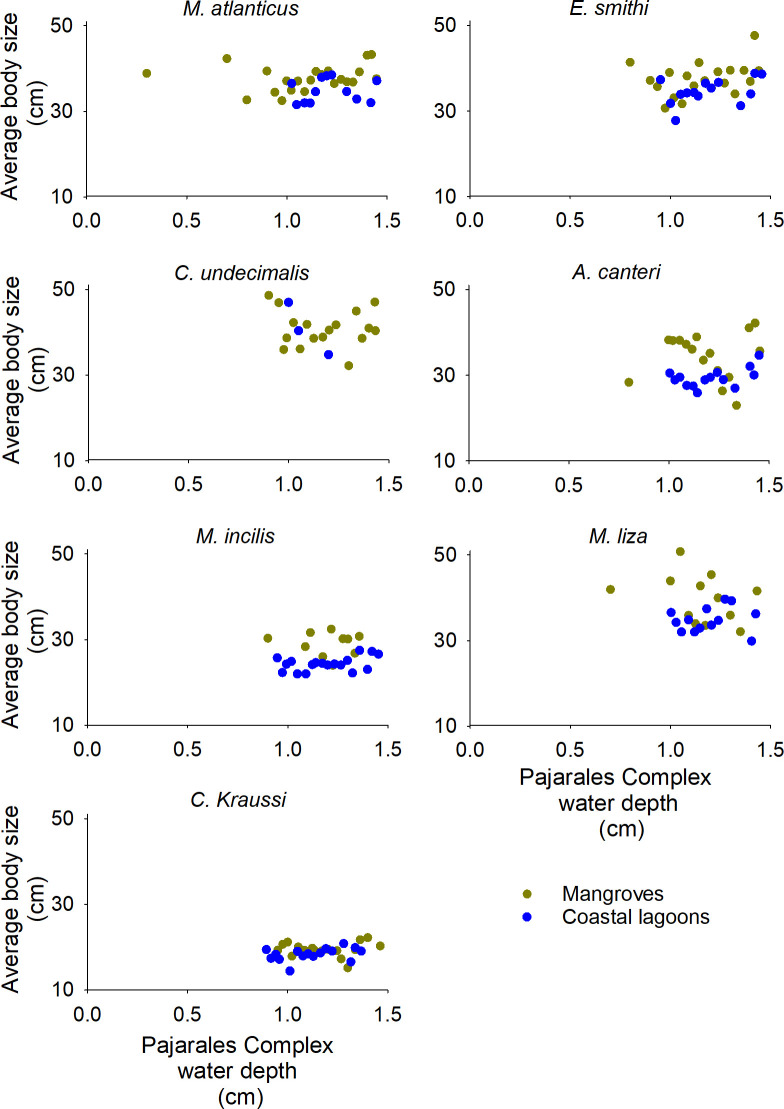
Average catch body size of seven fish species of the Pajarales complex *vs*. water depth.

For *E*. *smithi*, the utilization of mangroves was only significantly greater than that of coastal lagoons in adults and during La Niña phases (Mann-Whitney U, *p* < 0.05). In *M*. *atlanticus*, *C*. *undecimalis*, and *E*. *smithi*, larger adults were found in mangroves under wetter ENSO conditions and with low water depths usually recorded in drier ENSO phases (Fig 6 and S2 Fig, S1 Table).

For *E*. *plumieri*, the utilization of mangroves was higher than that of coastal lagoons across all body sizes and ENSO conditions (Mann–Whitney U, *p* < 0.05), except for 21–30 cm adults and during El Niño phases. Mangrove utilization decreased by 69% from La Niña to El Niño, but such changes did not significantly affect habitat utilization (Table 3).

The mangrove and coastal lagoon utilization of *A*. *canteri* was significantly affected by body size (Table 3), *i*.*e*., juveniles and smaller adults preferred coastal lagoons, while larger adults preferred mangroves (Mann-Whitney U, *p* < 0.05 in all cases). On average, larger fish were present in mangroves with lower water depths, and progressively smaller fish were found in coastal lagoons with drier ENSO conditions (Fig 6 and S2 Fig).

The utilization of coastal lagoons by *M*. *incilis* and *M*. *liza* was higher than that of mangroves across all body size classes and ENSO conditions (Mann-Whitney U, *p* < 0.05), except for larger adults and during El Niño in *M*. *incilis*, whose utilization of mangroves was influenced by ENSO conditions and their interaction with body size (Table 3, S2 Fig); it grew simultaneously with drier ENSO conditions and larger body sizes, reaching its highest value (61%) for larger adults during El Niño. In *M*. *incilis* and *M*. *liza*, habitat utilization was significantly affected by size (Table 3), with coastal lagoon utilization declining for larger sizes. In agreement with these results, the relationship between water depth and average body size shows that, on average, larger adults of these species are present in mangroves with lower water depths usually found under drier ENSO conditions.

In *C*. *mapale*, the utilization of coastal lagoons was also higher than that of mangroves for all body size classes and ENSO conditions (Mann-Whitney U, *p* < 0.05), except for juveniles and during neutral phases.

### Habitat utilization by freshwater fish species in response to size and climate variability

*O*. *niloticus* exhibited a higher utilization of mangroves because it was absent from coastal lagoons in all body sizes and across all ENSO conditions. The habitat utilization of *C*. *kraussi* was affected by size (Table 3); juveniles showed a higher utilization of coastal lagoons, while larger adults exhibited a higher utilization of mangroves (Mann-Whitney U, *p* < 0.05 in all cases).

The habitat utilization of *M*. *muyscorum* significantly changed according to ENSO conditions and their interaction with body size: juveniles were only present in coastal lagoons during La Niña, and they were only found in mangroves during neutral and El Niño phases (Fig 4 and S3 Fig). In contrast, larger adults showed a 32% higher use of mangroves during La Niña, compared to a 62% higher use of coastal lagoons during El Niño. Therefore, the average body size increased in mangroves with wetter conditions, and the relative mangrove utilization increased with drier conditions (S1 Table). Considering all size classes, the use of mangroves was only greater than that of coastal lagoons during neutral ENSO phases (Mann-Whitney U, *p* < 0.05).

In *P*. *magdalenae* and *H*. *malabaricus*, the higher habitat utilization of mangroves was not affected by body size or ENSO phases (Kruskal Wallis test, *p* > 0.05 in all cases). Mangroves did not exhibit a higher utilization during El Niño and La Niña, or by juveniles of both species (Mann-Whitney U, *p* > 0.05) due to low replicates. *i*.*e*., the absence of fish in one Pajarales zone.

In *T*. *magdalenae*, the utilization of coastal lagoons was higher than that of mangroves across all size classes and ENSO conditions (Mann-Whitney U, *p* < 0.05). However, mangrove utilization was significantly influenced by ENSO conditions and their interaction with body size, and it only occurred under neutral conditions, increasing from 3% in juveniles to 9% in adults.

## Discussion

### Habitat utilization by estuarine fish species in response to body size, climate variability, and water depth

The high dependence of the tarpon (*M*. *atlanticus*) and the snook (*C*. *undecimalis*) on mangroves agrees with several literature records. In a worldwide review, *C*. *undecimalis* was considered a highly mangrove-affiliated species, while sites with a high degree of mangrove edge accounted for a large number of *M*. *atlanticus* occurrences [69, 70]. Mangroves are recognized as nurseries for these species, corroborating our findings with regard to *M*. *atlanticus* and *C*. *undecimalis* juveniles’ greater preference for mangroves.

The preference of the ladyfish (*E*. *smithi*) for mangroves is consistent with a study showing the absence of *E*. *saurus* in disturbed mangroves when compared to pre-disturbed and restored sites (prior to 2010, *E*. *smithi* was usually identified as *E*. *saurus* in the Western Atlantic) [48, 71]. In the CGSM, *E*. *smithi* individuals caught during the day exhibited a relatively low or partial digested stomach contents, suggesting a nocturnal predatory behavior [48]. The encircling gillnets used in this study to estimate habitat utilization were set up during the day. Thus, the high mangrove utilization of *E*. *smithi* adults may indicate the use of this habitat as daytime resting and refuge sites.

In *M*. *atlanticus*, *C*. *undecimalis*, and *E*. *smithi*, the increasing utilization of larger body sizes in mangroves with drier ENSO conditions or lower water depths could indicate a predator avoidance strategy. Wading birds, which are common fish predators in mangrove environments, have high foraging success in shallow waters, as well as a greater preference for sites with sparse or intermediate vegetation density [72, 73]. Likewise, it has been found that some fish species avoid shallow waters far from cover or increase their use of cover at lower water depths [74, 75]. Consistently, predatory risk would increase for *M*. *atlanticus*, *C*. *undecimalis*, and *E*. *smithi*, which have relatively large bodies, in coastal lagoons at low inundation levels, as happens during El Niño. In such conditions, they would be easily located by predators. Increased mangrove utilization with larger body sizes and drier conditions has also been found in southwestern Florida for *Lutjanus griseus* and *Haemulon Sciurus*, which was attributed an expansion of habitat breadth with ontogeny and to a strategy to avoid predation [6].

The high mangrove utilization values found for *E*. *plumieri* agree with its plasticity regarding diet and habitat utilization. During El Niño, in several localities of the CGSM, this species exhibits a slightly higher utilization of mangroves than coastal lagoons [13]. Meanwhile, in the non-estuarine seascapes of Guadeloupe Island, *E*. *plumieri* utilizes and feeds in mangroves and seagrasses [26]. Although our records show *E*. *plumieri’s* greater preference for mangroves in comparison with coastal lagoons in the Pajarales Complex, this species ranked first and second in density and biomass in the main CGSM lagoon before 1998 [76, 77]. The collapse of oyster banks between 1996 and 2000, leading to that of the bivalve *Mytillopsis sallei*, the main food source of *E*. *plumieri* in coastal lagoons, drove a decline of this species in the system [76]. Currently, *E*. *plumieri* ranks fifth and twelfth, respectively, regarding encircling gillnet catch frequency in the main lagoon of CGSM and in the coastal lagoons of the Pajarales Complex. However, despite the decline in dominance, the generalized diet of this species [26, 49] may have favored the utilization of mangroves and its persistence in CGSM.

*A*. *Canteri’s* high utilization of mangroves and coastal lagoons is consistent with other studies carried out in the CGSM [13]. The shift in preference from coastal lagoons to mangroves found for larger body sizes is likely associated with the aforementioned predator avoidance strategy. Progressively larger fish are being recorded in mangroves at lower water depths, and smaller fish have been observed in coastal lagoons under drier conditions, suggesting that the former are avoiding coastal lagoons when the water depth is low in order to escape predation. The habitat preference changes of larger *A*. *canteri* individuals have also been reported in the Gulf of Urabá, another estuarine system in Colombia [78]. In such reports, the utilization of mangroves was higher in small juveniles (6.5–19.2 cm), which feed on the copepods of *R*. *mangle* prop roots, and mangrove utilization by larger body sizes decreased in response to diet shifts. These findings appear to contradict our results. However, it should be noted that assessing habitat utilization by *A*. *canteri* fish smaller than 15 cm was not possible in our study, and that the study conducted in the Gulf of Urabá did not evaluate habitat utilization as a function of climate variability or water depth. This suggests that *A canteri’s* habitat selection is highly dynamic and influenced by dietary shifts and predator avoidance behaviors in mangrove estuarine systems.

The high coastal lagoon utilization of *M*. *incilis*, *M*. *liza*, and *C*. *mapale* found in this work agrees with a study carried out in the CGSM, close to the Sevilla River mouth, albeit only for *M*. *incilis* [50], as *M*. *liza*, and *C*. *mapale* showed a higher utilization of the zones adjacent to mangroves. Such differences could be associated with habitat occupation (*M*. *liza*) and habitat reproduction requirements (*C*. *mapale*). The Lebranche mullet (*M*. *liza*) may be partially occupying the niche of the Nile tilapia (*O*. *niloticus*), which exhibited a low catch frequency in the zone adjacent to the mangroves of the Sevilla River mouth (5.7%) but usually dominates this transition zone. In fact, seine nets are employed in this ecotone between mangroves and coastal lagoons to capture Nile tilapia, accounting for 55% of its catch frequency. The similar diets of *O*. *niloticus* and *M*. *liza* (Table 1) suggest the latter’s high utilization of the coastal lagoons in the Pajarales Complex, as the zones adjacent to mangroves are occupied by the former. On the other hand, mangrove creeks are important reproduction sites for Cathorops species [79], whose habits may explain *C*. *mapale’s* high utilization of the zones adjacent to the mangroves of the Sevilla River mouth.

The increasing mangrove utilization by *M*. *incilis* and *M*. *liza* of larger body sizes under drier ENSO conditions, or the presence of larger fish in mangroves at lower water depths, could be related to a predator avoidance strategy, as explained earlier for *M*. *Atlanticus*, *C*. *undecimalis*, and *E*. *smithi*. Likewise, other observations suggest that predator avoidance is a habitat selection mechanism for *M*. *incilis* and *M*. *liza*. A relatively high utilization of mangroves by these species was observed in several localities of the CGSM during a moderate to strong El Niño episode [13], suggesting a high utilization of mangroves under low inundation conditions. In addition, fishermen describe *M*. *incilis* as moving into mangroves during low tides.

The results obtained, showing *C*. *mapale* juveniles’ similar utilization of mangrove and coastal lagoons, in addition to the fact that mainly juveniles are found in mangroves during La Niña phases (S2 Fig), are consistent with reports indicating that mangrove creeks are important reproduction sites for Cathorops species during wet periods [79]. On the other hand, the higher utilization of coastal lagoons by *C*. *mapale* under dry conditions agrees with previous findings in the CGSM [13].

Fish in mangroves are, on average, 1.5 cm larger than those in coastal lagoons due to the slightly larger gillnet mesh size used in mangrove habitats. This constantly larger mesh size does not affect the results demonstrating an increase in mangrove utilization during El Niño, nor does it impact the increased utilization of larger fish species in shallow water depths for *M*. *atlanticus*, *E*. *smithi*, *C*. *undecimalis*, *A*. *canteri*, M. incilis and *M*. *liza*

### Habitat utilization by freshwater fish species in response to body size and climate variability

The studied freshwater species are commercially relevant in the CGSM, and several of them are also common in other estuarine environments of the Colombian Caribbean [80–82]. However, with the exception of *O*. *niloticus*, the ecology of freshwater fish in tropical coastal lagoons has been less studied in comparison with that of estuarine fish [50, 83]. This work is one of the first to address the cross-habitat utilization of freshwater species within a flood plain lagoon.

The unique presence of *O*. *niloticus* in mangroves agrees with previous reports for the CGSM, which indicate its very low utilization of coastal lagoons; the Nile tilapia is more frequent in the ecotone between mangroves and coastal lagoons [50]. Similarly, other *Oreochromis* species exhibit a higher utilization of the ecotone between vegetation and coastal lagoons, as well as of vegetated habitats compared to unvegetated ones [84]. The use of vegetated littoral zones by *Oreochomis* species has been linked to seeking protection from predators [85, 86].

The increase in mangrove utilization by *C*. *kraussii* of larger body sizes may be related to ontogenetic dietary shifts, not to a predator avoidance strategy, since this species does not show a clear relationship between water depth and higher mangrove utilization by larger individuals. On the contrary, this species changes from an omnivorous diet to a fully piscivore one above 19 cm in the Ciénaga Grande de Lorica, Colombia [87]. We also found that, around the same size, its mangrove utilization increases.

*M*. *muyscorum* and *P*. *magdalenae* are migratory fish that spawn in main rivers and use coastal lagoon systems as nurseries during rainy seasons [88]. In addition, it has been suggested that these species use coastal lagoons as shelter and feeding areas, but no specific studies have assessed their cross-habitat utilization [83]. The changing habitat preferences of juveniles and adults of *M*. *muyscorum* in response to climate variability suggests that both mangroves and coastal lagoons play a role in the species’ reproduction and adult habitats. On the other hand, *H*. *malabaricus* has been widely described as a predator species associated with vegetation [89–91]. Our results, showing these three species’ great preference for mangroves, are consistent with previous studies on *H*. *malabaricus* and constitute the first report of *M*. *muyscorum* and *P*. *magdalenae’s* high utilization of mangrove-associated habitats within tropical coastal lagoon environments.

*T*. *magdalenae* is an endemic species of the Magdalena River basin that migrates from high to low watershed areas during dry seasons, presumably to reduce its risk of predation [92]. This species has been scarcely studied, and its preference for coastal lagoons has not been reported elsewhere. *T*. *magdalenae* has a predominantly zooplanktivorous diet [56], and its substantial utilization of coastal lagoons suggests that zooplankton is primarily exploited in this habitat. On the other hand, it is not clear why individuals of this species, particularly adults, increase their mangrove utilization only during neutral ENSO phases; more studies are necessary to better understand its cross-habitat utilization.

### Future studies

The Nile tilapia (*O*. *niloticus*) showed a preference for mangroves across all conditions and development stages, yet it is frequently captured in the CGSM’s transition zone between mangroves and coastal lagoons [50], a habitat not addressed in this work. It is relevant for future studies to assess the differential utilization of ecotones by estuarine and freshwater fish species [5].

The high utilization of a habitat does not necessarily indicate trophic utilization, and the fishing gear used in this work was deployed during daytime. Thus, although this study shows several tendencies in the habitat preferences of estuarine and freshwater species, future studies should focus on mechanisms of mangrove utilization. This includes conducting isotope analyses and assessing habitat utilization during nighttime, in order to better understand the entire scope of cross-habitat utilization by CGSM fish species and the mangrove-fishery linkage.

## Conclusions

The high values and increments in mangrove utilization by fish species of the Pajarales Complex in the CGSM are related to different mechanisms, not always to the nursery function. Some species exhibit a high or increasing utilization of mangroves, presumably as consequence of feeding plasticity or dietary shifts. On the other hand, the relatively low inundation usually observed during El Niño events increases the risk of predation and, consequently, the utilization of mangroves by larger fish. Therefore, predator avoidance is a key driver of mangrove utilization and habitat selection in the eutrophic-hypereutrophic turbid waters of the Pajarales Complex.

Water depth in the Pajarales Complex is influenced by climate variability fluctuations, but also by seasonal and daily changes in response to fluvial inputs and tides. All temporal scales of water level fluctuations are expected to generate habitat selection responses by fish in deltaic systems to limit predation.

This study’s findings support recent suggestions regarding the importance of conserving and managing habitat complexity in estuarine environments to sustain fish populations [13, 14]. However, the CGSM’s area of fringe and riverine mangroves, which are subject to tidal or fluvial inundation, is only 1.2% of that occupied by coastal lagoons. Thus, protection–and even the expansion–of these key mangrove types is relevant for the management of CGSM fisheries.

## Supporting information

S1 Table Correlation analysis between the intensity of climate variability (evaluated through the ONI), the average body size, and the relative habitat utilization of fish species. (DOCX)

S1 Fig Average salinity and dissolved oxygen ±SE in the Pajarales Complex zones during the ENSO phases between 2001 and 2019. (TIF)

S2 Fig Violin plots of the body sizes of the main estuarine species caught in different habitats across ENSO phases in the Pajarales Complex of CGSM. M: mangrove, CL: coastal lagoon.
(TIF)

S3 Fig Violin plots of the body sizes of the main freshwater species caught in different habitats across ENSO phases in the Pajarales Complex of the CGSM. M: mangrove, CL: coastal lagoon.
(TIF)

## References

[1] Carrasquilla-Henao M, Juanes F. Mangroves enhance local fisheries catches: a global meta-analysis. Fish and Fisheries. 2017;18(1):79–93.

[2] Serafy JE, Shideler GS, Araújo RJ, Nagelkerken I. Mangroves enhance reef fish abundance at the Caribbean regional scale. PLoS One. 2015;10(11):1–15. doi: 10.1371/journal.pone.0142022 26536478 PMC4633132

[3] Sandoval-Londoño LA, Leal-Flórez J, Blanco-Libreros JF, Mancera-Pineda JE, Delgado-Huertas A, Polo-Silva CJ. Stable-isotope analysis reveals sources of organic matter and ontogenic feeding shifts of a mangrove-dependent predator species, New Granada sea catfish, Ariopsis canteri. J Fish Biol. 2020;(May). 10.1111/jfb.14404 32445234

[4] Thayer GW, Colby DR, Hettler WF. Utilization of the red mangrove prop root habitat by fishes in south Florida. Mar Ecol Prog Ser. 1987;35:25–38.

[5] Sheaves M, Baker R, Nagelkerken I, Connolly RM. True Value of Estuarine and Coastal Nurseries for Fish: Incorporating Complexity and Dynamics. Estuaries and Coasts. 2015;38(2):401–14.

[6] Faunce CH, Serafy JE. Nearshore habitat use by gray snapper (Lutjanus griseus) and bluestriped grunt (Haemulon sciurus): Environmental gradients and ontogenetic shifts. Bull Mar Sci. 2007;80(3):473–95.

[7] Rönnbäck P. The ecological basis for economic value of seafood production supported by mangrove ecosystems. Ecological Economics. 1999;29(2):235–52.

[8] Laegdsgaard P, Johnson C. Why do juvenile fish utilise mangrove habitats? J Exp Mar Biol Ecol. 2001;257(2):229–53. doi: 10.1016/s0022-0981(00)00331-2 11245878

[9] Le DQ, Tanaka K, Hii YS, Sano Y, Nanjo K, Shirai K. Importance of seagrass-mangrove continuum as feeding grounds for juvenile pink ear emperor Lethrinus lentjan in Setiu Lagoon, Malaysia: Stable isotope approach. J Sea Res. 2018;135(February):1–10.

[10] Le DQ, Fui SY, Tanaka K, Suratman S, Sano Y, Shirai K. Feeding habitats of juvenile reef fishes in a tropical mangrove-seagrass continuum along a Malaysian shallow-water coastal lagoon. Bull Mar Sci. 2020;96(3):469–86.

[11] Ramirez-Martínez GA, Castellanos-Galindo GA, Krumme U. Tidal and Diel Patterns in Abundance and Feeding of a Marine-Estuarine-Dependent Fish from Macrotidal Mangrove Creeks in the Tropical Eastern Pacific (Colombia). Estuaries and Coasts. 2016;39(4):1249–61.

[12] Lapointe BE, Tomasko DA, Matzie WR. Eutrophication and trophic state classification of seagrass communities in the Florida Keys. Bull Mar Sci. 1994;54(3):696–717.

[13] Carrasquilla-Henao M, Rueda M, Juanes F. Fish habitat use in a Caribbean mangrove lagoon system. Estuar Coast Shelf Sci. 2022 Nov 5;278.

[14] Marley GSA, Deacon AE, Phillip DAT, Lawrence AJ, Marley G. Mangrove or mudflat: prioritising fish habitat for conservation in a turbid tropical estuary.

[15] Sierra-Rozo O, Santos-Martínez A, Arturo AP. Prospección ecológica del manglar y praderas marinas como hábitats de cría para peces arrecifales en san Andrés isla, caribe insular colombiano. Boletín de Investigaciones Marinas y Costeras. 2012;41(2):375–98.

[16] Worthington TA, zu Ermgassen PSE, Friess DA, Krauss KW, Lovelock CE, Thorley J, et al. A global biophysical typology of mangroves and its relevance for ecosystem structure and deforestation. Sci Rep. 2020;10(1):1–11. 32887898 10.1038/s41598-020-71194-5 PMC7473852

[17] Huijbers CM, Nagelkerken I, Debrot AO, Jongejans E, Ecology S, August N, et al. Geographic coupling of juvenile and adult habitat shapes spatial population dynamics of a coral reef fish. Ecology. 2013;94(8):1859–70. doi: 10.1890/11-1759.1 24015529

[18] Nagelkerken I, Sheaves M, Baker R, Connolly RM. The seascape nursery: A novel spatial approach to identify and manage nurseries for coastal marine fauna. Fish and Fisheries. 2015;16(2):362–71.

[19] Cai W, Ng B, Geng T, Wu L, Santoso A, McPhaden MJ. Butterfly effect and a self-modulating El Niño response to global warming. Nature. 2020;585:68–73. 32879502 10.1038/s41586-020-2641-x

[20] Possamai B, Vieira JP, Grimm AM, Garcia AM. Temporal variability (1997–2015) of trophic fish guilds and its relationships with El Niño events in a subtropical estuary. Estuar Coast Shelf Sci. 2018 Mar 5;202:145–54.

[21] Belarmino E, Francisco de Nóbrega M, Grimm AM, da Silva Copertino M, Vieira JP, Garcia AM. Long-term trends in the abundance of an estuarine fish and relationships with El Niño climatic impacts and seagrass meadows reduction. Estuar Coast Shelf Sci. 2021 Oct 31;261.

[22] Mol JH, Resida D, Ramlal JS, Cor &, Becker R Effects of El Niño-related drought on freshwater and brackish-water fishes in Suriname, South America. Environ Biol Fishes. 2000;59:429–40.

[23] Blanco JA, Narváez Barandica JC, Viloria EA. ENSO and the rise and fall of a tilapia fishery in northern Colombia. Fish Res. 2007;88(1–3):100–8.

[24] Sandoval-Londoño LA, Leal-Flórez J, Blanco-Libreros JF. Linking mangroves and fish catch: A correlational study in the southern Caribbean Sea (Colombia). Bull Mar Sci. 2020;96(3):415–29.

[25] Kurth BN, Peebles EB, Stallings CD. Atlantic Tarpon (Megalops atlanticus) exhibit upper estuarine habitat dependence followed by foraging system fidelity after ontogenetic habitat shifts. Estuar Coast Shelf Sci. 2019;225(June):106248.

[26] Vaslet A, Bouchon-navaro Y, Harmelin-vivien M, Lepoint G, Louis M, Bouchon C. Foraging habits of reef fishes associated with mangroves and seagrass beds in a Caribbean lagoon: A stable isotope approach. Cienc Mar. 2015;41:217–32.

[27] Kimirei IA, Nagelkerken I, Mgaya YD, Huijbers CM. The Mangrove Nursery Paradigm Revisited: Otolith Stable Isotopes Support Nursery-to-Reef Movements by Indo-Pacific Fishes. PLoS One. 2013;8(6). doi: 10.1371/journal.pone.0066320 23776658 PMC3680401

[28] Restrepo JC, Ortíz JC, Pierini J, Schrottke K, Maza M, Otero L, et al. Freshwater discharge into the Caribbean Sea from the rivers of Northwestern South America (Colombia): Magnitude, variability and recent changes. J Hydrol (Amst) [Internet]. 2014;509(December 2017):266–81. Available from: 10.1016/j.jhydrol.2013.11.045

[29] Blanco JA, Viloria EA, Narváez B. JC. ENSO and salinity changes in the Cienaga Grande de Santa Marta coastal lagoon system, Colombian Caribbean. Estuar Coast Shelf Sci. 2006;66(1–2):157–67.

[30] Ibarra KP, Gómez MC, Viloria EA, Arteaga E, Cuadrado I, Martínez MF, et al. Monitoreo de las condiciones ambientales y los cambios estructurales y funcionales de las comunidades vegetales y los recursos pesqueros durante la rehabilitación de la Ciénaga Grande de Santa Marta. 2015;140 p. + apendix.

[31] Sánchez-Núñez DA, Rodríguez-Rodríguez JA, Mancera Pineda JE. Effects of climate variability and hydrological rehabilitation measures on long-term mangrove trajectories: From reproduction to recruitment and landscape cover changes. Journal of Applied Ecology. 2023 Dec 1;60(12):2508–20.

[32] Carrasquilla-Henao M, Ban N, Rueda M, Juanes F. The mangrove-fishery relationship: A local ecological knowledge perspective. Mar Policy. 2019;108(August).

[33] Vargas-Morales M, Sánchez-Núñez D, Amaya E, Contreras, Andrea Sánchez-Maldonado J, Acosta A, Pérez D, et al. Valoración Integral de los Principales Bienes y Servicios ecosistémicos provistos por los ecosistemas de manglar. Elementos Técnicos y Generación de Capacidad para el Ordenamiento y Manejo de los espacios y recursos marinos, costeros e insulares de Colombia. Santa Marta, Colombia; 2013.

[34] Carrasquilla-Henao M, González Ocampo HA, Luna González A, Rodríguez Quiroz G. Mangrove forest and artisanal fishery in the southern part of the Gulf of California, Mexico. Ocean Coast Manag. 2013;83:75–80.

[35] Botero L, Salzwedel H. Rehabilitation of the Ciénaga Grande de Santa Marta, a mangrove-estuarine system in the Caribbean coast of Colombia. Ocean Coast Manag. 1999;42:243–56.

[36] Rivera-Monroy VH, Twilley RR, Mancera E, Alcantara-Eguren A, Castañeda-Moya E, Casas Monroy O, et al. Aventuras y desventuras en Macondo: rehabilitación de la Ciénaga Grande de Santa Marta, Colombia. Ecotropicos. 2006;19(2):72–93.

[37] Vivas-Aguas LJ, Espinosa LF, Parra LG. Identificación de fuentes terrestres de contaminación y cálculo de las cargas contaminantes en el área de influencia de la Ciénaga Grande de Santa Marta, Caribe Colombiano. Boletín de Investigaciones Marinas y Costeras. 2013;42(1):7–30.

[38] Ricaurte C, Bastidas M, Briceño F, Caicedo F, Arbeláez J, Herrera S, et al. Estudio integral de la CGSM. Ampliación de la fase II A. Modelo batimétrico de caños y ciénagas secundarias de interconexión con el río Magdalena. Fase II B1 modelo hidro-sedimentológico conceptual del complejo lagunar CGSM. Santa Marta, Colombia; 2018.

[39] INVEMAR. Monitoreo de las condiciones ambientales y los cambios estructurales y funcionales de las comunidades vegetales y de los recursos pesqueros durante la rehabilitación de la Ciénaga Grande de Santa Marta. Informe Técnico. Santa Marta; 2019.

[40] INVEMAR. Monitoreo de las condiciones ambientales y los cambios estructurales y funcionales de las comunidades vegetales y de los recursos pesqueros durante la rehabilitación de la Ciénaga Grande de Santa Marta. Informe Técnico Final 2023, Volumen 22. Santa Marta 196 p. [Internet]. 2023. Available from: www.invemar.org.co

[41] Botero L, Mancera-Pineda JE. Síntesis de los cambios de origen antrópico ocurridos en los últimos 40 años en la Ciénaga de Santa Marta (Colombia). Rev Acad Colomb Cienc Exactas Fis Nat [Internet]. 1996;20(78):465–74. Available from: http://www.accefyn.org.co/revista/Vol_20/78/465-474.pdf

[42] Twilley RR, Rivera-Monroy VH, Chen R, Botero L. Adapting an ecological mangrove model to simulate trajectories in restoration ecology. Mar Pollut Bull. 1998;37(8–12):404–19.

[43] Cardona CJG, Moreno JY, Contreras A, Sanchez-Nuñez DA, Moreno NA, Guerrero D, et al. Accounting of marine and coastal ecosystems at the Ramsar Site, Estuarine Delta System of the Magdalena River, Ciénaga Grande de Santa Marta, Colombia. One Ecosystem. 2023;8.

[44] Aguilera M. Habitantes del agua: El complejo lagunar de la Ciénaga Grande de Santa Marta. Documentos de trabajo sobre economía regional. Cartagena: Banco de la República. Centro de Estudios Económicos Regionales (CEER)-CARTAGENA; 2011. 49 p.

[45] Restrepo JD, Kjerfve B. Magdalena river: Interannual variability (1975–1995) and revised water discharge and sediment load estimates. J Hydrol (Amst). 2000;235(1–2):137–49.

[46] Peterson MS, Slack WT, Brown-Peterson NJ, Mcdonald JL. Reproduction in Nonnative Environments: Establishment of Nile Tilapia, Oreochromis niloticus, in Coastal Mississippi Watersheds. Copeia. 2004;(4):842–9.

[47] Harrington RW, Harrington ES. Food of larval and young tarpon, Megalops atlantica. Copeia. 1960;311–9.

[48] Santos Martínez A, Arboleda S. Aspectos Biológicos Y Ecológicos Del Macabi Elops Saurus Linnaeus (Pisces: Elopidae) en La Ciénaga Grande de Santa Marta y Costa Adyacente, Caribe Colombiano. Boletín de Investigaciones Marinas y Costeras-INVEMAR. 1993;22:77–96.

[49] Arenas-Granados P, Acero A. Organización trófica de las mojarras (Pisces: Gerreidae) de la Ciénaga Grande de Santa Marta (Caribe Colombiano). Rev Biol Trop. 1992;40(3):287–302.

[50] Gámez B, Morón E, Fuentes J. Descripción del hábito alimentario de doce especies de peces asociados a la Ciénaga Grande de Santa Marta, Colombia. Boletín de Investigaciones Marinas y Costeras. 2014;43(1):23–42.

[51] Blewett DA, Hensley RA, Stevens PW. Feeding Habits of Common Snook, Centropomus undecimalis, in Charlotte Harbor, Florida. Gulf Caribb Res. 2006 Jan 1;18.

[52] Osorio D. Ecología trófica de Mugil curema, M. incilis y M. liza (pisces: Mugilidae) en la Ciénaga Grande de Santa Marta, Caribe Colombiano. I. Análisis cualitativo y cuantitativo. Boletín de Investigaciones Marinas y Costeras. 1988;18:113–26.

[53] Casas JY, Lozano-Largacha Y, Lara TR. Contribución a la ecología trófica del dentón Leporinus muyscorum (Steindachner 1902) en la Ciénaga la Grande, cuenca media del río Atrato, Colombia. Revista Institucional Universidad Tecnológica del Chocó. 2007;26:4–8.

[54] Ramírez C A, Gabriel Pinilla A. Hábitos alimentarios, morfometría y estados gonadales de cinco especies de peces en diferentes períodos climáticos en el río Sogamoso (Santander, Colombia). Acta Biolo Colomb. 2012;17(2):241–58.

[55] Carvalho LN, Velasquez CH, Sul VS. Alimentação de Hoplias malabaricus (Bloch, 1794)(Osteichthyes, Erythrinidae) no rio Vermelho, Pantanal Sul Mato-Grossense. Revista Brasileira de Zoociencias. 2002;4(2):227–36.

[56] Morales J, García-Alzate CA. Ecología trófica y rasgos ecomorfológicos de Triportheus magdalenae (Characiformes: Triportheidae) en el embalse El Guájaro, cuenca baja del río Magdalena, Colombia. Rev Biol Trop. 2018;66(3):1208.

[57] Narváez JC, Rueda M, Viloria E, Blanco J, Romero JA, Newmark F. Manual del Sistema de Información Pesquera del INVEMAR (SIPEIN V.3.0): Una herramienta para el diseño de sistemas de manejo pesquero. INVEMAR (Serie de documentos generales del INVEMAR No. 18). Santa Marta; 2005.

[58] INVEMAR. Base de Datos. Sistema de Información Pesquera de INVEMAR-SIPEIN-CGSM. Consulta de Variables de desempeño Pesquero. 2021.

[59] INVEMAR. Monitoreo de las condiciones ambientales y los cambios estructurales y funcionales de las comunidades vegetales y de los recursos pesqueros durante la rehabilitación de la Ciénaga Grande de Santa Marta. Informe Técnico. Santa Marta, Colombia; 2018.

[60] Rueda M, Blanco J, Narváez JC, Viloria EA, Beltrán C. Coastal Fisheries of Colombia. In: Salas S, Chuenpagdee R, Charles A, Seijo JC, editors. Coastal fisheries of Latin America and the Caribbean FAO Fisheries and Aquaculture Technical Paper No 544. Rome: FAO; 2011. p. 430.

[61] Puentes V, Polo CJ, Roldán AM, Zuluaga P. A. Artes y Métodos de Pesca en Colombia. Serie Recursos Pesqueros de Colombia–AUNAP 2014. 2014.

[62] NOAA/ National Weather Service. National Centers for Environmental Prediction. Historical El Niño/La Niña episodes (1950-present) [Internet]. 2024 [cited 2024 May 14]. Available from: http://origin.cpc.ncep.noaa.gov/products/analysis_monitoring/ensostuff/ONI_v5.php

[63] INVEMAR. Sistema de Información Ambiental Marina de Colombia–SIAM. Base de datos del sistema de información de calidad ambiental marina de la REDCAM. Información de calidad de aguas de variables Físico/Químicas en estaciones ubicadas en el complejo Pajarales de la Ciénaga Grande de Santa Marta—CGSM, departamento de Magdalena. Serie de datos 2000 hasta 2020. Santa Marta; 2024.

[64] INVEMAR-MINAMBIENTE. Cuarto Informe técnico. Convenio Interadministrativo 659 de 2017. Santa Marta, Colombia; 2018.

[65] Scheirer F, Ray WS, Hare N. The analysis of ranked data derived from completely randomized factorial designs. International Biometric Society. 1976;32(2):429–34. 953139

[66] Sokal RR, Rohlf FJ. Biometry: the principles and practice of statistics in biological research. 3rd edition. 3rd ed. New York: WH Freeman & Company; 1995. 887 p.

[67] INVEMAR. Monitoreo de las condiciones ambientales y los cambios estructurales y funcionales de las comunidades vegetales y de los recursos pesqueros durante la rehabilitación de la Ciénaga Grande de Santa Marta. Informe Técnico. 2017.

[68] INVEMAR. Monitoreo hidrosedimentológico de la Ciénaga Grande de Santa Marta 2018–2030. Informe técnico de actividades. Santa Marta; 2021.

[69] Zugelter A. Nursery Habitat Characteristics of Juvenile Tarpon, Megalops atlanticus, in the northern Indian River Lagoon, FL. Florida Institute of Technology; 2019.

[70] Zu Ermgassen PSE, Grove T, Nagelkerken I. Global affiliation of juvenile fishes and invertebrates with mangrove habitats. Bull Mar Sci. 2020;96(3):403–14.

[71] Lewis RR, Gilmore RG. Important considerations to achieve successful mangrove forest restoration with optimum fish habitat. Bull Mar Sci. 2007;80(3):823–37.

[72] Lantz SM, Gawlik DE, Cook MI. The Effects of Water Depth and Emergent Vegetation on Foraging Success and Habitat Selection of Wading Birds in the Everglades. Waterbirds [Internet]. 2011;439–77. Available from: https://bioone.org/journals/Waterbirds

[73] Ruiz-Guerra C, Echeverry-Galvis MÁ. Prey consumed by wading birds in mangrove swamps of the Caribbean coast of Colombia. J Nat Hist. 2019 Aug 11;53(29–30):1823–36.

[74] Harvey BC, White JL. Axes of fear for stream fish: water depth and distance to cover. Environ Biol Fishes. 2017 May 1;100(5):565–73.

[75] Gibson R J, Erkinaro J. The influence of water depths and inter-specific interactions on cover responses of juvenile Atlantic salmon. Ecology of Freshwater Fish. 2009;18:629–39.

[76] Viloria-Maestre E, Arturo AP, Blanco J. El colapso de la pesquería de la mojarra rayada Eugerres plumieri (pisces: Gerreidae) en la ciénaga grande de santa marta: ¿causas pesqueras, ambientales o biológicas? Boletín de Investigaciones Marinas y Costeras. 2012;41(2):399–428.

[77] Rueda M, Defeo O. Spatial structure of fish assemblages in a tropical estuarine lagoon: Combining multivariate and geostatistical techniques. J Exp Mar Biol Ecol. 2003;296(1):93–112.

[78] Sandoval-Londoño L, Leal-Flórez J, Blanco-Libreros JF, Taborda-Marín A. Hábitos alimenticios y aspectos del uso del hábitat por el chivo cabezón Ariopsis sp. (aff. assimilis) (Siluriformes: Ariidae), en una laguna costera neotropical (Ecorregión Darién, Colombia). Actualidades Biológicas. 2015;37(102):295–306.

[79] Giarrizzo T, Krumme U. Spatial differences and seasonal cyclicity in the intertidal fish fauna from four mangrove creeks in a salinity zone of the Curuçá estuary, north Brazil. Bull Mar Sci. 2007;80(3):739–54.

[80] Román-Valencia C, Acero A. Notas sobre las comunidades de peces del norte de Antioquia (Colombia). Boletín de Investigaciones Marinas y Costeras. 1992;21:117–25.

[81] Arango-Sánchez LB, Correa-Herrera T, Correa-Rendón JD. Fish diversity in estuarine habitats of the Atrato river delta, gulf of urabá. Boletin Cientifico del Centro de Museos. 2019;23(1):191–207.

[82] Leal-Flórez J, Rueda M, Wolff M. Role of the non-native fish Oreochromis niloticus in the long-term variations of abundance and species composition of the native ichthyofauna in a Caribbean estuary. Bull Mar Sci. 2008;82(3):365–80.

[83] Jiménez-Segura LF, Palacio J, Leite R. River flooding and reproduction of migratory fish species in the Magdalena River basin, Colombia. Ecol Freshw Fish. 2010;19(2):178–86.

[84] Økland F, Hay CJ, Næsje TF, Chanda B, Thorstad EB. Movements of, and habitat utilisation by, threespot tilapia Oreochromis andersonii (Teleostei: Cichlidae) in the Upper Zambezi River, Namibia. Afr J Aquat Sci. 2007;32(1):35–8.

[85] Donnelly BG. A preliminary survey of Tilapia nurseries on Lake Kariba during 1967/68. Hydrobiologia. 1969;34:195–206.

[86] Russell DJ, Thuesen PA, Thomson FE. A review of the biology, ecology, distribution and control of Mozambique tilapia, Oreochromis mossambicus (Peters 1852) (Pisces: Cichlidae) with particular emphasis on invasive Australian populations. Rev Fish Biol Fish. 2012;22(3):533–54.

[87] Lerma MM, Ramos LM. Hábitos alimentarios de la mojarra amarilla Caquetaia kraussii (Steindachner, 1878) en la Ciénaga Grande de Lorica, Colombia. BSc Thesis. 2020.

[88] Jiménez-Segura LF, Galvis-Vergara G, Cala-Cala P, García-Alzate CA, López-Casas S, Ríos-Pulgarín MI, et al. Freshwater fish faunas, habitats and conservation challenges in the Caribbean river basins of north-western South America. J Fish Biol. 2016;89(1):65–101. doi: 10.1111/jfb.13018 27401480

[89] Prado CPA, Gomiero LM, Froehlich O. Spawning and parental care in Hoplias malabaricus (Teleostei, Characiformes, Erythrinidae) in the southern Pantanal, Brazil. Brazilian Journal of Biology. 2006;66(2 B):697–702. 10.1590/s1519-69842006000400013 16906301

[90] Mazzoni R, Iglesias-Rios R. Distribution pattern of two fish species in a coastal stream in Southeast Brazil. Brazilian Journal of Biology. 2002;62(1):171–8. 10.1590/s1519-69842002000100019 12185917

[91] Gois KS, Antonio RR, Gomes LC, Pelicice FM, Agostinho AA. The role of submerged trees in structuring fish assemblages in reservoirs: Two case studies in South America. Hydrobiologia. 2012;685(1):109–19.

[92] Valdelamar-Villegas JC. Apuntes sobre la importancia ecológica, ambiental y social de la arenca Triportheus magdalenae (Steindachner, 1878). Un ejemplo de endemismo invisibilizado. Intropica. 2018;152–65.

