# Supplementary material for: Cross-habitat utilization of fish in a tropical deltaic system as a function of climate variability and body size: Are mangroves fish nurseries in a tropical delta?
Source: PLoS One. 2024 Aug 16;19(8):e0308313. doi: 10.1371/journal.pone.0308313 (PMC11329160; doi:10.1371/journal.pone.0308313)
Supplement: S1 Table — (DOCX) [file pone.0308313.s001.docx]

|  | **Average body size** | | **Relative habitat utilization** | |
| --- | --- | --- | --- | --- |
|  | **Mangroves** | **Coastal lagoons** | **Mangroves** | **Coastal lagoons** |
| *M. atlanticus* | -0.45* | -0.42^ns^ | 0.05^ns^ | -0.05^ns^ |
| *E. smithi* | -0.33* | -0.46* | -0.18^ns^ | 0.18^ns^ |
| *E. plumieri* | 0.04^ns^ | -0.58^ns^ | -0.30^ns^ | 0.30^ns^ |
| *A. canteri* | -0.03^ns^ | -0.32^ns^ | -0.32^ns^ | 0.32^ns^ |
| *C. undecimalis* | -0.42* | 0.50^ns^ | 0.18^ns^ | -0.18^ns^ |
| *M. incilis* | 0.18^ns^ | -0.25^ns^ | -0.22^ns^ | 0.22^ns^ |
| *M. liza* | -0.02^ns^ | -0.35^ns^ | -0.04^ns^ | 0.04^ns^ |
| *C. mapale* | 0.61^ns^ | 0.03^ns^ | -0.17^ns^ | 0.17^ns^ |
| *O. niloticus* | -0.36^ns^ |  | 0.38^ns^ |  |
| *C. kraussi* | -0.18^ns^ | 0.16^ns^ | 0.35^ns^ | -0.35^ns^ |
| *M. muyscorum* | -0.51* | 0.40^ns^ | 0.56* | -0.56* |
| *P. magdalenae* | -0.25^ns^ | NED | 0.41^ns^ | -0.41^ns^ |
| *H. malabaricus* | -0.49^ns^ | NED | 0.22^ns^ | -0.22^ns^ |
| *T. magdalenae* | -0.20^ns^ | 0.13^ns^ | 0.39^ns^ | -0.39^ns^ |

**p* < 0.05, ^ns^*p* > 0.05, NED: not enough data for the correlation analysis
