# Supplementary figures and images for: Cross-habitat utilization of fish in a tropical deltaic system as a function of climate variability and body size: Are mangroves fish nurseries in a tropical delta?
Source: PLoS One. 2024 Aug 16;19(8):e0308313. doi: 10.1371/journal.pone.0308313 (PMC11329160; doi:10.1371/journal.pone.0308313)

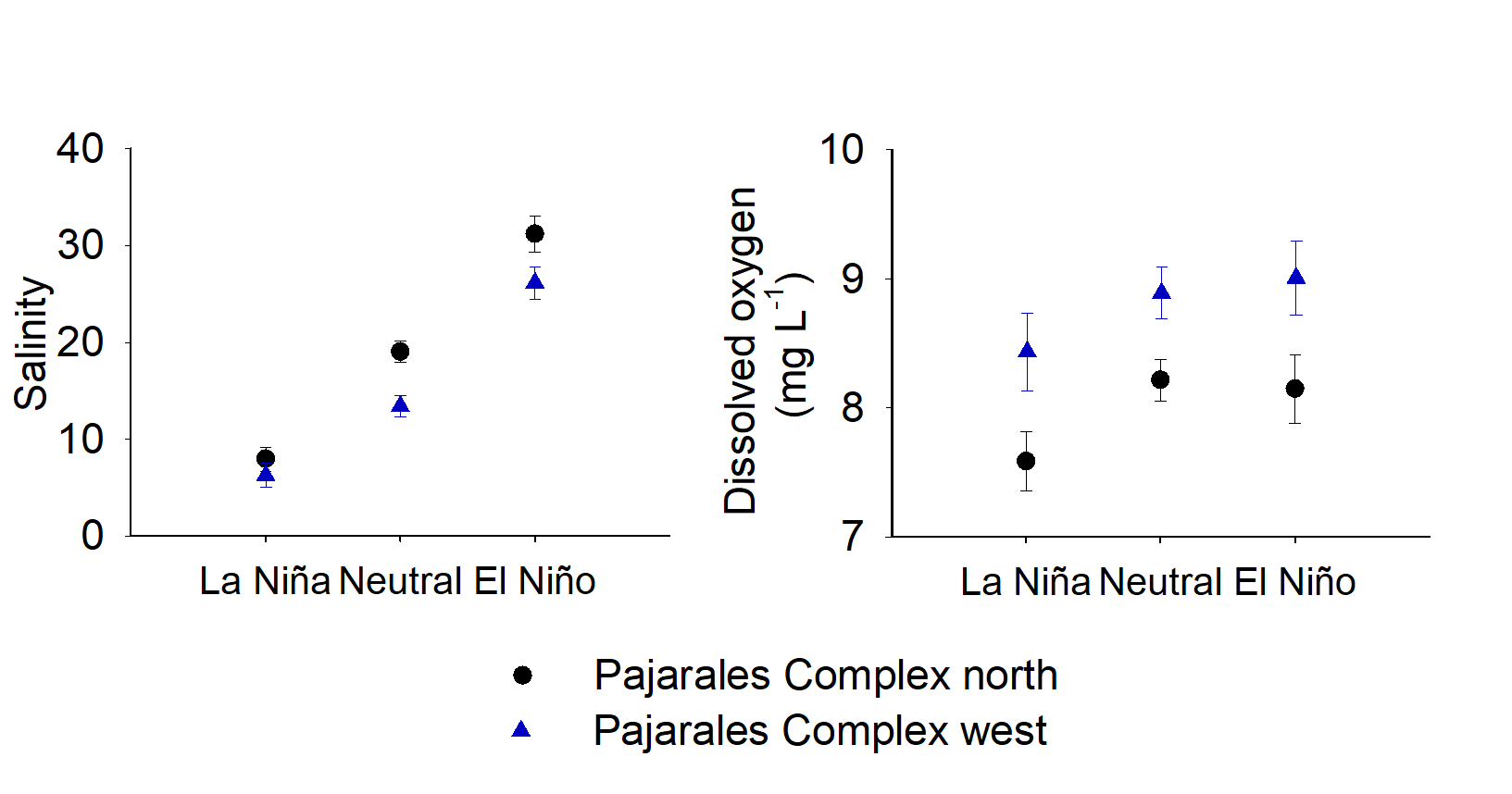

Supplement: S1 Fig — (TIF) [file pone.0308313.s002.tif]

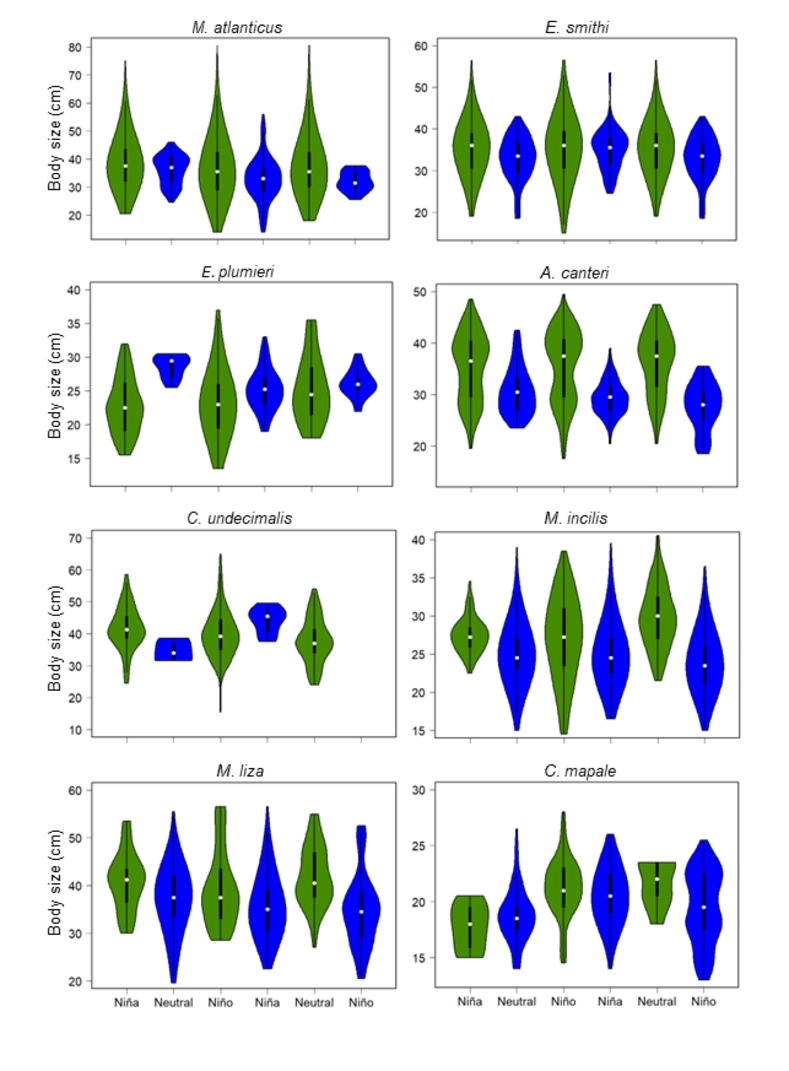

Supplement: S2 Fig — M: mangrove, CL: coastal lagoon. (TIF) [file pone.0308313.s003.tif]

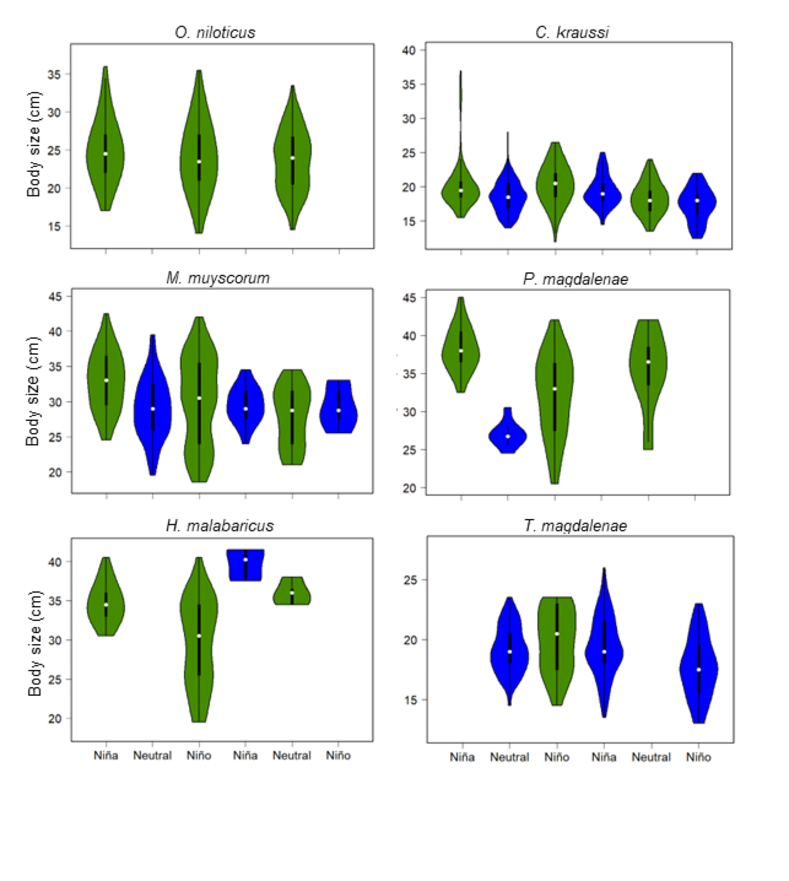

Supplement: S3 Fig — M: mangrove, CL: coastal lagoon. (TIF) [file pone.0308313.s004.tif]
